# A Highly Substituted Ring-Fused 2-Pyridone Compound Targeting PrfA and the Efflux Regulator BrtA in Listeria monocytogenes

**DOI:** 10.1128/mbio.00449-23

**Published:** 2023-04-25

**Authors:** Hasan Tükenmez, Pardeep Singh, Souvik Sarkar, Melike Çakır, Ana H. Oliveira, Cecilia Lindgren, Karolis Vaitkevicius, Mari Bonde, A. Elisabeth Sauer-Eriksson, Fredrik Almqvist, Jörgen Johansson

**Affiliations:** a Department of Chemistry, Umeå University, Umeå, Sweden; b Umeå Centre of Microbial Research (UCMR), Umeå University, Umeå, Sweden; c Molecular Infection Medicine, Sweden (MIMS), Umeå University, Umeå, Sweden; d Department of Molecular Biology, Umeå University, Umeå, Sweden; e QureTech Bio, Umeå, Sweden; University of Washington

**Keywords:** 2-pyridones, BrtA, *Listeria monocytogenes*, PrfA, antibacterial, antibiotic

## Abstract

Listeria monocytogenes is a facultative Gram-positive bacterium that causes listeriosis, a severe foodborne disease. We previously discovered that ring-fused 2-pyridone compounds can decrease virulence factor expression in Listeria by binding and inactivating the PrfA virulence activator. In this study, we tested PS900, a highly substituted 2-pyridone that was recently discovered to be bactericidal to other Gram-positive pathogenic bacteria, such as Staphylococcus aureus and Enterococcus faecalis. We show that PS900 can interact with PrfA and reduce the expression of virulence factors. Unlike previous ring-fused 2-pyridones shown to inactivate PrfA, PS900 had an additional antibacterial activity and was found to potentiate sensitivity toward cholic acid. Two PS900-tolerant mutants able to grow in the presence of PS900 carried mutations in the *brtA* gene, encoding the BrtA repressor. In wild-type (WT) bacteria, cholic acid binds and inactivates BrtA, thereby alleviating the expression of the multidrug transporter MdrT. Interestingly, we found that PS900 also binds to BrtA and that this interaction causes BrtA to dissociate from its binding site in front of the *mdrT* gene. In addition, we observed that PS900 potentiated the effect of different osmolytes. We suggest that the increased potency of cholic acid and osmolytes to kill bacteria in the presence of PS900 is due to the ability of the latter to inhibit general efflux, through a yet-unknown mechanism. Our data indicate that thiazolino 2-pyridones constitute an attractive scaffold when designing new types of antibacterial agents.

## INTRODUCTION

Listeria monocytogenes is a Gram-positive foodborne pathogen that can cause severe infections (listeriosis) in different animals and in humans. Listeriosis can appear as a sporadic infection or as a disease outbreak, with mortality rates of up to 30% (1). L. monocytogenes uses several virulence factors to traverse different barriers (intestinal, placental, and blood-brain), as well as to survive inside the gut.

L. monocytogenes is a great concern for the food industry due to its ability to withstand and grow at a wide range of temperatures (−0.4°C to 45°C) and pH levels (4.3 to 9.6), as well as high salt concentrations (up to 20%) (2, 3). Since L. monocytogenes is able to form biofilms, it is difficult to eradicate once it contaminates a food processing environment, despite being forced into harsh conditions (4, 5). Among other food preservatives, nitrite (NO_2_, E250) and nitrate (NO_3_, E252) are often added to food products, such as meat, to reduce L. monocytogenes growth (6).

Being a foodborne pathogen, L. monocytogenes enters the host through consumption of contaminated food. Once ingested, the bacterium enters the intestinal tract, where it able to reside for a couple of days before passing the intestinal barrier. In a human population study, L. monocytogenes was detected in the stool of 2.1% of asymptomatic individuals (7). Interestingly, L. monocytogenes has the ability to colonize the gallbladders of mice, guinea pigs, and turkeys (8–11), and the bacterium can use the gallbladder as a reservoir from which it can be shed into the gastrointestinal tract (12). Therefore, it has been suggested that L. monocytogenes might be responsible for human cholecystitis (13, 14). This is further underlined by its ability to grow in gallbladder bile (15). When L. monocytogenes is exposed to bile, several genes alter their expression, among them a TetR-like regulator named BrtA (bile-regulated transcription factor A) (16). BrtA acts as a transcriptional repressor, blocking the expression of multidrug transporters, such as MdrT. Upon interaction with cholic acid, the protein is displaced from the DNA, thus allowing the expression of MdrT and removal of cholic acid from the cytoplasm (16). L. monocytogenes also expresses a bile salt hydrolase (Bsh) and a bile exclusion protein (BilE) (17–19). Bsh and BilE are important for the survival of the bacterium in the intestinal tract, especially in the duodenum at a low bile pH. The proteins appear to work in concert with other factors, such as OpuC (a carnitine transporter) and BetL (a betaine uptake system) (20).

When traversing the intestinal barrier, mainly using goblet cells, L. monocytogenes enters epithelial cells guided by the adhesin internalin A, which recognizes E-cadherin (21, 22). Once inside the host cell, the bacterium is able to escape the phagosome and spread to the adjacent cells using listeriolysin O (LLO) and ActA, respectively (22). The latter recruits the Arp2/3 complex, leading to a massive polymerization of actin at the pole of the bacterium (an actin comet tail), thereby facilitating bacterial movement and protrusion to the next cell (22).

Most virulence factors in L. monocytogenes, including LLO and ActA, are controlled by a single regulator, PrfA (23). PrfA is a transcriptional activator that recognizes and binds a stretch of bases (PrfA box) located at the operator region of PrfA-regulated genes. The binding of PrfA to the PrfA box is enhanced by the coactivator glutathione, which in its reduced form binds PrfA at a tunnel site located in each monomer of the PrfA dimer (24, 25). The PrfA-glutathione interaction repositions the helix-turn-helix (HTH) motif of the protein, thus facilitating DNA binding.

Interestingly, ring-fused 2-pyridones can bind to the same part of the PrfA protein as glutathione, but with a 1,000-fold-higher affinity (26). This PrfA–2-pyridone interaction prevents the HTH motif from adopting the DNA binding conformation. As a result, ring-fused 2-pyridones are efficient virulence blockers that are able to suppress virulence gene expression and cell infection almost completely (26, 27).

In this study, we have examined the activity of a second-generation 2-pyridone (PS900) in Listeria monocytogenes. PS900 was identified when a library of second-generation 2-pyridones was screened for antibacterial activity against several Gram-positive bacteria (28). Compared to 2-pyridones that inactivate PrfA, the second-generation 2-pyridones are more functionalized, having additional substituents and being equipped with a carbon tail via a phenoxy linker. In this study, we show that this substituted 2-pyridone is able not only to interact with PrfA and repress virulence gene expression equally as well as the previous generation of 2-pyridones but also to inhibit L. monocytogenes growth. The isolation of suppressor mutations and *in vitro* experiments provide evidence that the more functionalized 2-pyridones interact with BrtA and inhibit the protein from binding its cognate DNA sequence, in a manner similar to the way in which cholic acid disrupts BrtA-DNA binding. As a consequence, the BrtA–2-pyridone interaction leads to derepressed expression of MdrT. The simultaneous addition of cholic acid and the 2-pyridone leads to bacterial lethality, possibly connected to the decreased efflux observed in 2-pyridone-treated bacteria. Finally, we provide evidence that the 2-pyridone is able to potentiate nitrite-mediated killing of L. monocytogenes 50-fold.

## RESULTS

### A new generation of ring-fused 2-pyridones can inhibit virulence gene expression by binding and inactivating PrfA.

**FIG 1:**
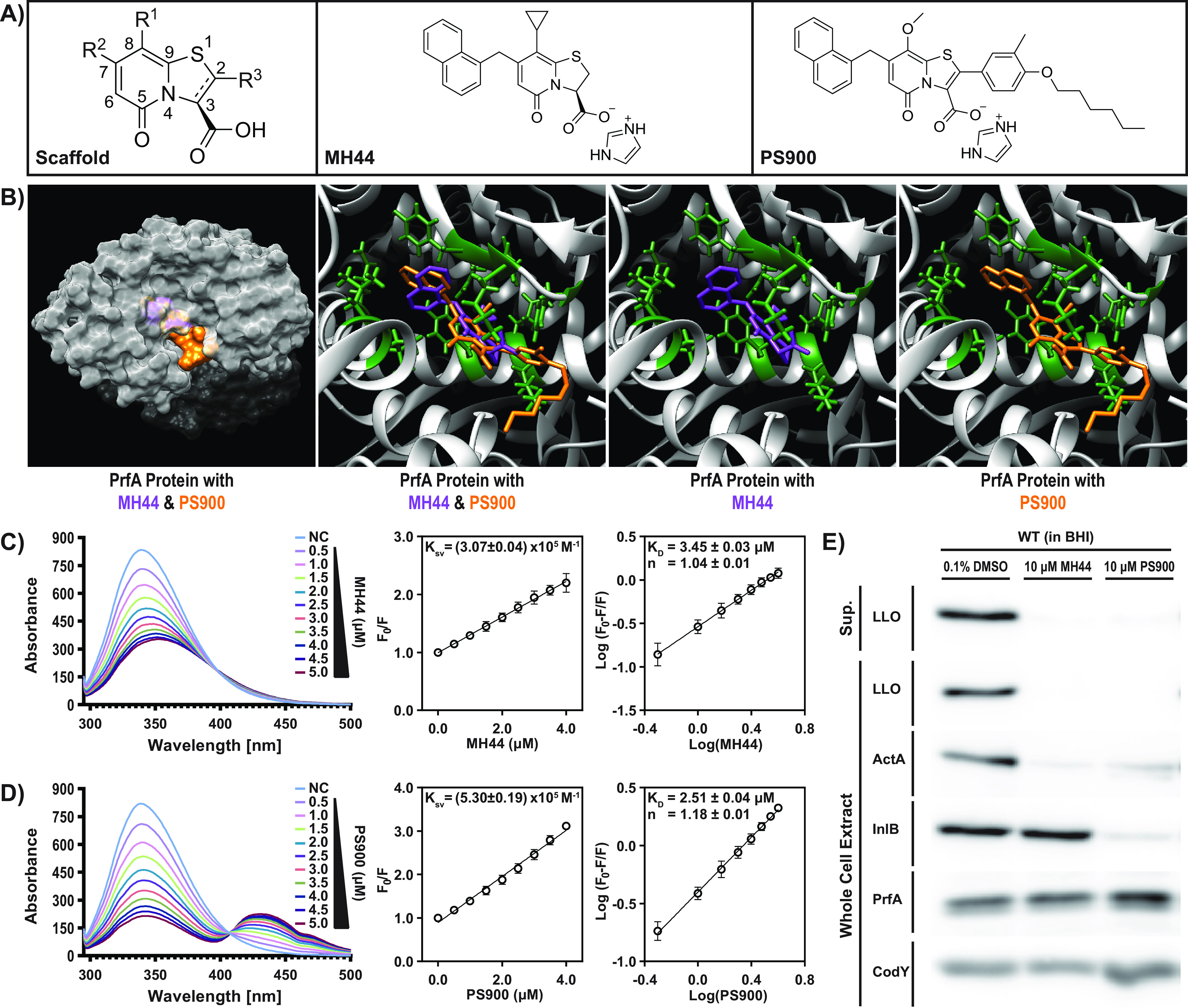
PS900 interacts with PrfA and reduces the expression of virulence factors in L. monocytogenes. (A) Chemical structures of the scaffold, MH44 (C10-IMD), and PS900. (B) Superposition of the X-ray crystal conformation of MH44 (purple) (PDB code 5F1R [26]) and the docked conformation of PS900 (orange) in the binding domain of virulence regulator PrfA from L. monocytogenes (green). (C, D) Fluorescence-quenching spectra of PrfA in the presence of MH44 (C) or PS900 (D). A concentration of 5 μM PrfA in PBS solution was excited at 290 nm, and quenching of fluorescence was recorded in the presence of various concentrations of the compounds (0.5 to 5 μM). Stern-Volmer plots of decreases in the fluorescence of PrfA in the presence of various concentrations of compounds were used to determine the dynamic quenching rate constant, *K*_sv_ (calculated from the slope of the line). Logarithmic plots of relative fluorescence quenching of PrfA against logarithmic concentrations of compounds were used to determine *K_D_* (calculated from the intersection of the line with the *y* axis) and the number of binding sites, *n* (calculated from the slope of the line). Error bars indicate standard deviations calculated from three individual experiments. NC, No compound. (E) Virulence gene expression in the WT strain in the presence of MH44 or PS900. The protein extracts were isolated from secreted fractions (Sup., supernatant) and whole-cell fractions of bacteria grown in the presence of 10 μM MH44, 10 μM PS900, or 0.1% DMSO (control) for 3 h at 37°C. The levels of LLO, ActA, InlB, PrfA, and CodY (control) proteins were visualized by Western blotting using specific antibodies. The images are representative of three individual experiments.

We previously observed that the binding of various ring-fused 2-pyridones to the transcriptional regulator of virulence in L. monocytogenes, PrfA, decreased the ability of the protein to interact with its DNA motif (26, 27). This PrfA–2-pyridone interaction also reduced virulence gene expression and infectivity in cell lines and a chicken embryo model (27, 29). Structural analysis identified 2-pyridones (represented here by MH44) (Fig. 1A) as binding a central part of the PrfA protein, leading to a displacement of the helix-turn-helix motif that was unfavorable for DNA binding. In order to generate more-efficient compounds, we took advantage of 2-pyridones carrying a long carbon tail at position C-3 (represented here by PS900) (Fig. 1A). We have previously shown that such 2-pyridones show antibacterial activity against other Gram-positive bacteria (28). Docking PrfA with PS900, using the previously characterized PrfA-MH44 structure as a model, showed that PS900 fits within the 2-pyridone binding pocket identified previously (Fig. 1B) (26, 27). To examine whether PS900 interacts with PrfA, fluorescence-quenching experiments were conducted with purified PrfA protein and 2-pyridones (Fig. 1C and D; Fig. S1 in the supplemental material). Our results show that PS900 is able to interact with PrfA with an affinity comparable to that of the previously characterized PrfA-MH44 complex.

10.1128/mbio.00449-23.1 FIG S1 Fluorescence-quenching spectra of PrfA protein in the presence of DMSO (control). (A) Fluorescence quenching of various concentrations of MH44 (0.5 to 5.0 μM) in the absence of protein. (B) Fluorescence quenching of various concentrations of PS900 (0.5 to 5.0 μM) in the absence of protein. (C) A concentration of 5 μM PrfA in PBS solution was excited at 290 nm, and quenching of fluorescence was recorded in the presence of various concentrations of DMSO (0.2 to 2.0%). (D, E) Fluorescence-quenching spectra of BrtA protein in the presence of DMSO (control) (D) or MH44 (E). A concentration of 5 μM BrtA in PBS solution was excited at 290 nm, and quenching of fluorescence was recorded in the presence of various concentrations of DMSO (0.2 to 3.0%) or MH44 (0.5 to 5 μM). Download FIG S1, EPS file, 2.0 MB. Copyright © 2023 Tükenmez et al. 2023 Tükenmez et al. https://creativecommons.org/licenses/by/4.0/ This content is distributed under the terms of the Creative Commons Attribution 4.0 International license.

To assess whether the second-generation 2-pyridones could also affect virulence gene expression, bacteria were grown in BHI medium in the presence of 10 μM PS900. Our results showed that PS900 was able to repress the expression of the most prevalent virulence factors (LLO and ActA) at a level comparable to that of MH44 without affecting the levels of PrfA (Fig. 1E). Interestingly, unlike MH44, PS900 also repressed the expression of the adhesin InlB. The reason for this difference is unclear, but we do not exclude the possibility that it might involve activity of SigB, the alternative sigma factor in L. monocytogenes, which is important for *inlB* expression (30).

We conclude that the new generation of ring-fused 2-pyridones can interact with and reduce the expression of virulence genes in L. monocytogenes, possibly by inactivating PrfA, as previously shown for other 2-pyridones (26, 27).

### PS900 is bactericidal in defined medium (DM) but not in BHI medium.

In a previous study, we showed that PS900 had bacteriostatic activity for several Gram-positive pathogens at low micromolar concentrations (MICs between 0.39 and 3.12) (28) when the bacteria were grown in brain heart infusion (BHI) medium. We were therefore interested to examine whether PS900 could inhibit the growth of L. monocytogenes in a similar manner. Surprisingly, we were unable to detect inhibition of L. monocytogenes growth in BHI at concentrations of PS900 that blocked the growth of other Gram-positive pathogens (Fig. S2) (28). Instead, we only observed bacteriostatic activity of PS900 at high (80 μM) concentrations. Regardless of the PS900 concentration, we were unable to detect any bactericidal activity (Fig. S2).

10.1128/mbio.00449-23.2 FIG S2 Small growth inhibition of bacteria grown in BHI medium in the presence of PS900. Representative images showing the effects of MH44 and PS900 on bacterial growth and survival in BHI medium. Effects on bacterial growth (MICs) were scored by pellet formation and resazurin (blue)-to-resorufin (pink) conversion (see Materials and Methods). Bacterial survival (MBC) was scored by growth on regular BHI agar plates. The WT strain was incubated overnight in BHI medium with various concentrations of DMSO (control), MH44, or PS900 at 37°C. Samples were then spotted onto regular BHI agar plates, followed by 24 h of incubation at 37°C. The images are representatives of three individual experiments. Download FIG S2, EPS file, 5.6 MB. Copyright © 2023 Tükenmez et al. 2023 Tükenmez et al. https://creativecommons.org/licenses/by/4.0/ This content is distributed under the terms of the Creative Commons Attribution 4.0 International license.

**FIG 2:**
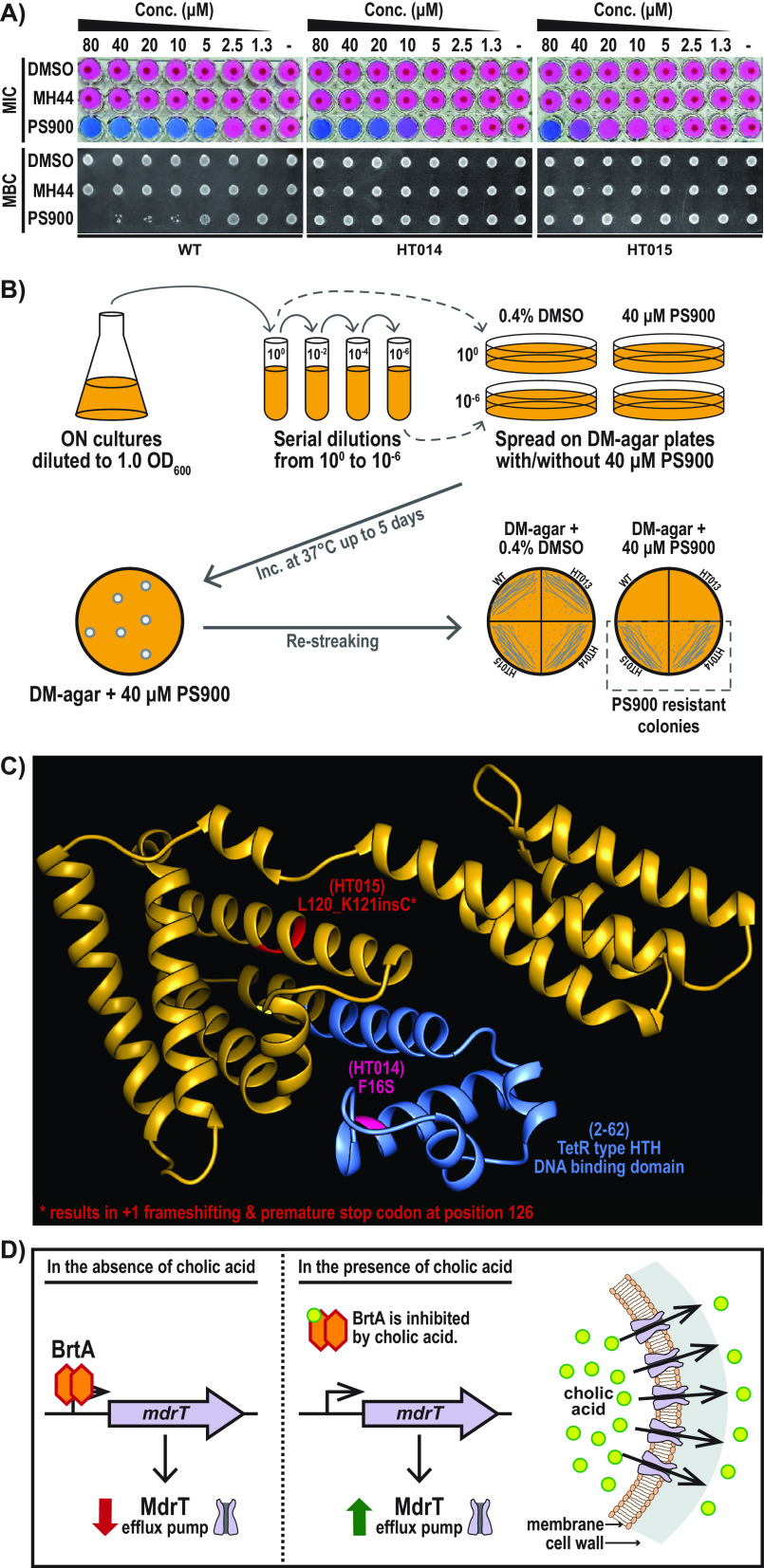
PS900 inhibits L. monocytogenes growth in defined medium. (A) Representative images showing the effect of PS900 on bacterial growth (MIC), scored by pellet formation and resazurin (blue)-to-resorufin (pink) conversion (see Materials and Methods). Bacterial survival (MBC, minimal bactericidal concentration) was scored by growth on BHI agar plates. The WT strain and PS900-resistant mutants (HT014 and HT015) were incubated overnight in defined medium with various concentrations of DMSO (control), MH44, or PS900 at 37°C (showing MICs). These samples were then spotted onto regular BHI agar plates, followed by 24 h of incubation at 37°C (showing MBCs). The images are representative of three individual experiments. (B) Schematic model depicting the PS900-resistant-mutant selection process. ON, overnight. (C) Predicted TetR-type HTH DNA binding domain (blue) and the amino acid substitutions found in the BrtA protein in the HT014 (pink) and HT015 (red) strains are highlighted on the AlphaFold-predicted structure of the BrtA protein (Fig. S3) (52, 53). (D) Schematic model depicting the regulation of MdrT efflux pump expression by transcriptional repressor BrtA in the absence or presence of cholic acid (16).

L. monocytogenes is a soil bacterium that occasionally becomes ingested and can infect a eukaryotic host. Being exposed to a plethora of environments, it has developed a number of mechanisms, such as efflux pumps, to survive harsh conditions (31). Such defense mechanisms are of different importance depending on the growth medium. L. monocytogenes shows a greater osmotic tolerance in a complex medium like BHI, due to BHI’s high levels of peptone compared to the level in a chemically defined medium (DM) (32). We thus were interested to examine whether PS900 could affect the growth or survival of L. monocytogenes in DM. Our results showed a bacteriostatic effect of PS900 at 5 μM, accompanied by a bactericidal activity at 10 μM, when L. monocytogenes was cultivated in DM (Fig. 2A).

10.1128/mbio.00449-23.3 FIG S3 AlphaFold structural model of L. monocytogenes BrtA protein. (A) AlphaFold model output of BrtA protein (https://alphafold.ebi.ac.uk/entry/Q8Y466) color coded by model confidence score (52, 53). (B) The expected positional error panel showing well-defined dark green patches for the two domains (highlighted in grey and green on the structure to the right). The weaker green shading between these two domains indicates that their relative orientation is not well defined. Download FIG S3, EPS file, 7.0 MB. Copyright © 2023 Tükenmez et al. 2023 Tükenmez et al. https://creativecommons.org/licenses/by/4.0/ This content is distributed under the terms of the Creative Commons Attribution 4.0 International license.

### Inactivation of the transcriptional repressor BrtA suppresses the antibacterial activity of PS900.

To further elucidate the mechanism by which PS900 reduces L. monocytogenes growth and viability, we set up a plate experiment to select for suppressor mutants able to grow in DM in the presence of 40 μM PS900 (Fig. 2B). Two such suppressor mutants were able to grow and survive also at high levels of PS900 (Fig. 2A and B) and were subjected to whole-genome sequencing (WGS) (Table S1A). Unlike the WT strain, both suppressor mutants carried mutations in the gene *lmo2589*, which encodes the transcriptional regulator BrtA (Fig. 2C; Table S1B). One of the suppressor mutants (HT014) carried a missense mutation in the *brtA* gene, leading to a phenylalanine-to-serine exchange at position 16 (*brtA*_F16S_), whereas the other suppressor mutant (HT015) had a +1 frameshift in the *brtA* gene, leading to a premature stop codon at position 126.

10.1128/mbio.00449-23.9 TABLE S1 (A) Details of Illumina sequence results. (B) SNP analysis of WT, HT014, and HT015 strains. Download Table S1, PDF file, 0.03 MB. Copyright © 2023 Tükenmez et al. 2023 Tükenmez et al. https://creativecommons.org/licenses/by/4.0/ This content is distributed under the terms of the Creative Commons Attribution 4.0 International license.

In L. monocytogenes, BrtA has been shown to control the expression of the multidrug transporter MdrT by acting as a repressor (Fig. 2D) (16). MdrT is an efflux pump that is important for the bacterium to survive elevated cholic acid (CA) levels. In the absence of CA, the BrtA protein binds to the operator region upstream from *mdrT*, thereby repressing the expression of the latter (16). When CA is present, it interacts with and inhibits the repressive activity of BrtA, thus alleviating *mdrT* expression. Elevated expression of the efflux pump MdrT allows the bacterium to efficiently decrease the intracellular concentration of CA (16).

### PS900 increases *mdrT* expression and allows bacteria to grow in the presence of CA.

**FIG 3:**
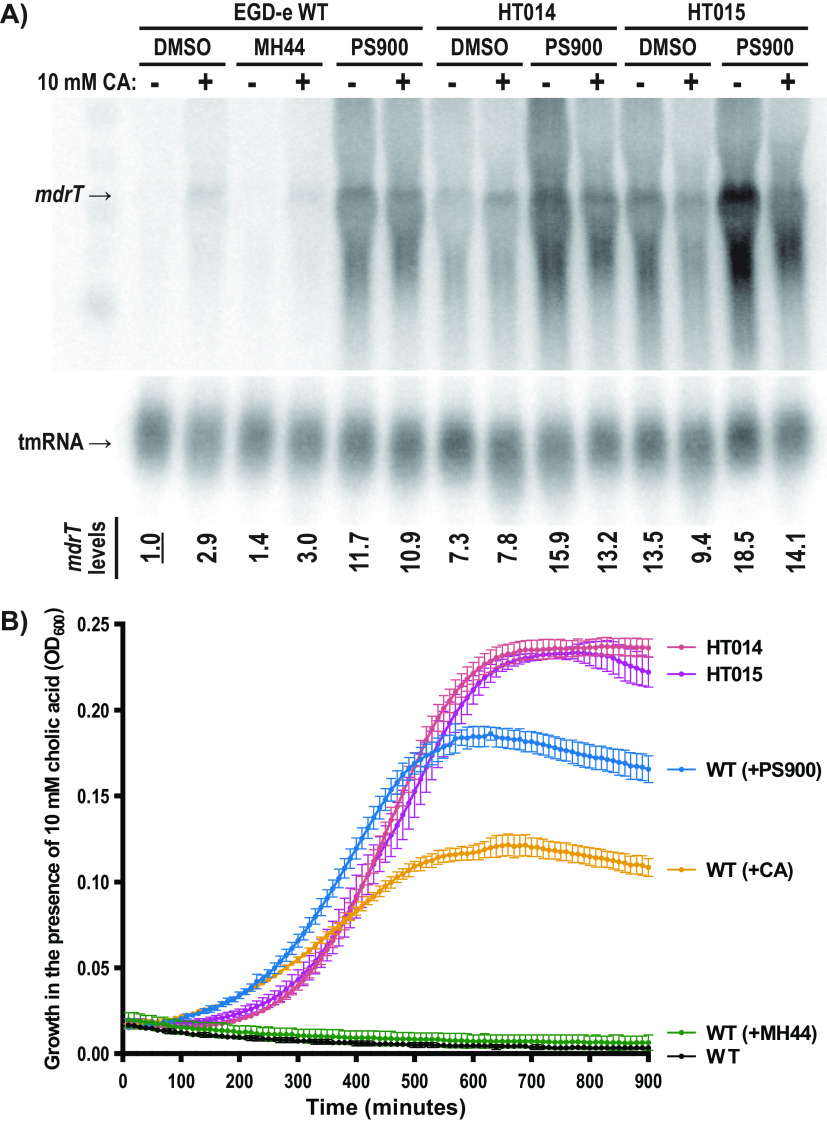
PS900 induces *mdrT* expression. (A) *mdrT* efflux pump expression in the presence of MH44, PS900, and/or cholic acid. The total RNA was isolated from the WT, HT014, and HT015 strains, which were grown in the presence of 10 μM MH44, 10 μM PS900, or 0.1% DMSO (control) for 2.5 to 3 h at 37°C in BHI medium. Then, the cultures were divided into two subcultures. One set was incubated for another 30 min at 37°C with 10 mM cholic acid, whereas the other set was incubated with DMSO (control). The levels of *mdrT* and tmRNA (control) were visualized by Northern blotting using specific probes. The *mdrT* levels from each sample were normalized to the tmRNA levels. The results for each sample were normalized to those of the WT strain grown in the presence of 0.1% DMSO without cholic acid. The images are representative of three individual experiments. (B) Growth of WT, HT014, and HT015 strains in BHI medium in the presence of 10 mM CA. Overnight WT, HT014, and HT015 cultures were washed and precultivated in BHI medium in the presence of 0.1% DMSO (control), 10 μM MH44, 10 μM PS900, or 10 mM CA for an hour at 37°C. These pretreated cultures were then diluted to an OD_600_ of 0.02 and grown for 15 h at 37°C. All measurements were performed in triplicates, and the results were plotted using GraphPad Prism (version 9.3.1). Error bars represent standard deviations of replicates.

Based on our results, we hypothesize that inactivated BrtA (as in the suppressor mutants) increases the expression of the MdrT efflux pump, thereby allowing bacterial growth in the presence of PS900 (Fig. 2). To challenge this idea and to further explore the role played by the compounds, WT and suppressor mutants were grown in the presence or absence of MH44 and PS900 before the addition of CA (or not) for half an hour. The experiments were performed in BHI to avoid effects caused by PS900-mediated growth inhibition. We found that the presence of CA induced the expression of *mdrT* in the WT strain, likely through CA interacting with and inactivating BrtA, as has been shown previously (Fig. 3A; Fig. S4) (16). The HT014 and HT015 suppressor mutants showed constitutive expression of *mdrT* regardless of the condition, in line with them carrying dysfunctional variants of the BrtA repressor. Interestingly, the presence of PS900 (but not MH44) resulted in increased *mdrT* expression in the WT strain (Fig. 3A; Fig. S4). The expression of *brtA* was low in the WT strain except when PS900 was added (Fig. S4). In contrast, the expression of *brtA* was high in the mutant strains, indicating an autoregulatory loop involving the BrtA repressor.

10.1128/mbio.00449-23.4 FIG S4 *mdrT* efflux pump expression in the presence of MH44, PS900, and/or cholic acid (Northern blots). (A) Individual replicates of the Northern blots depicting the levels of *mdrT* and tmRNA. The total RNA was isolated from the WT, HT014, and HT015 strains, which were grown in the presence of 10 μM MH44, 10 μM PS900, or 0.1% DMSO (control), respectively, for 2.5 to 3 h at 37°C. Then, the cultures were divided into two subcultures. One set was incubated for another 30 minutes at 37°C with 10 mM cholic acid, whereas the other set was incubated with DMSO (control). The levels of *mdrT*, *brtA*, and tmRNA (control) were visualized by Northern blotting using specific probes. The *mdrT* and *brtA* levels from each sample were normalized to the tmRNA levels. The results for each sample were normalized to those of the WT strain grown in the presence of 0.1% DMSO without cholic acid. (B, C) Plots representing the normalized *mdrT* (B) and *brtA* (C) levels of each biological replicate normalized to the expression of tmRNA as shown in panel A. Error bars indicate the standard deviations among the replicates. Download FIG S4, EPS file, 7.7 MB. Copyright © 2023 Tükenmez et al. 2023 Tükenmez et al. https://creativecommons.org/licenses/by/4.0/ This content is distributed under the terms of the Creative Commons Attribution 4.0 International license.

In light of the finding that PS900 can induce *mdrT* expression, we asked whether the increased level of *mdrT* in bacteria exposed to PS900 was sufficient to allow bacteria to grow in the presence of CA. To examine this, WT bacteria were preexposed (or not) to 10 mM CA, 10 μM PS900, or 10 μM MH44 for 1 h before regrowing the bacteria in the presence of 10 mM CA for 15 h. Preincubation with dimethyl sulfoxide (DMSO) (control) or MH44 completely abrogated bacterial growth after the addition of CA (Fig. 3B). In contrast, preincubation of the bacteria with CA or PS900 enabled growth at a level similar to that observed for the suppressor mutants HT014 and HT015, which already had derepressed *mdrT* expression (Fig. 3B).

Although both HT014 and HT015 had mutations in *brtA* and showed increased *mdrT* expression, explaining their increased resistance to CA and PS900, we sought to confirm this further and rule out effect(s) of other possible mutations. Plasmid constructs carrying the wild-type (WT) *brtA* (*brtA*_WT_) and the *brtA*_F16S_ genes were created and introduced into the WT, the HT014 mutant (having the *brtA*_F16S_ mutation on the chromosome), and the HT015 mutant (carrying a *brtA* frameshift mutation leading to a truncated BrtA protein), respectively. These constructs were inoculated into medium with or without CA and PS900, and their growth monitored (Fig. S5). We hypothesized that reintroducing a functional BrtA protein would cause a synthetic growth deficiency in the mutants in the presence of CA and PS900, provided that the ability of HT014 and HT015 to grow under the same conditions was indeed caused by mutations in the *brtA* gene. In agreement with this argument, the HT014 and HT015 strains carrying *brtA*_WT_ showed reduced growth ability (although not as low as that of the WT strain) in the presence of CA and PS900 compared to the growth of the same strains carrying the vector control pMK4 (Fig. S5). The reason why HT014 carrying BrtA_WT_ did not show a fully reduced growth rate (as in the WT strain) in the presence of either CA or PS900 could be that BrtA_F16S_ acts as a dominant-negative mutant protein, partially reducing BrtA_WT_’s activity. Reintroducing BrtA_F16S_ into the HT014 and HT015 strains resulted in slightly decreased growth rates compared to those of the vector controls. Our results thus suggest that an inactivated BrtA protein causes increased resistance of the HT014 and HT015 strains to CA and PS900, although we cannot exclude the possibility of other genetic differences contributing to the phenotype.

10.1128/mbio.00449-23.5 FIG S5 Growth of WT, HT014, and HT015 strains complemented with pMK4, pMK4-BrtA^WT^, or pMK4-BrtA^F16S^. (A, B) All strains were grown overnight in BHI medium with 10 μg/mL chloramphenicol at 37°C. Overnight cultures were diluted to an OD_600_ of 0.05 in BHI medium with 10 μg/mL chloramphenicol containing 0.1% DMSO without cholic acid (CA) (A) or 10 μM PS900 with 10 mM CA (B). The cultures were grown for 7.5 h at 37°C. All measurements were performed in triplicates, and the results were plotted using GraphPad Prism (version 9.3.1). Error bars represent standard deviations of replicates. Download FIG S5, EPS file, 1.7 MB. Copyright © 2023 Tükenmez et al. 2023 Tükenmez et al. https://creativecommons.org/licenses/by/4.0/ This content is distributed under the terms of the Creative Commons Attribution 4.0 International license.

### PS900 interacts with and inactivates BrtA.

**FIG 4:**
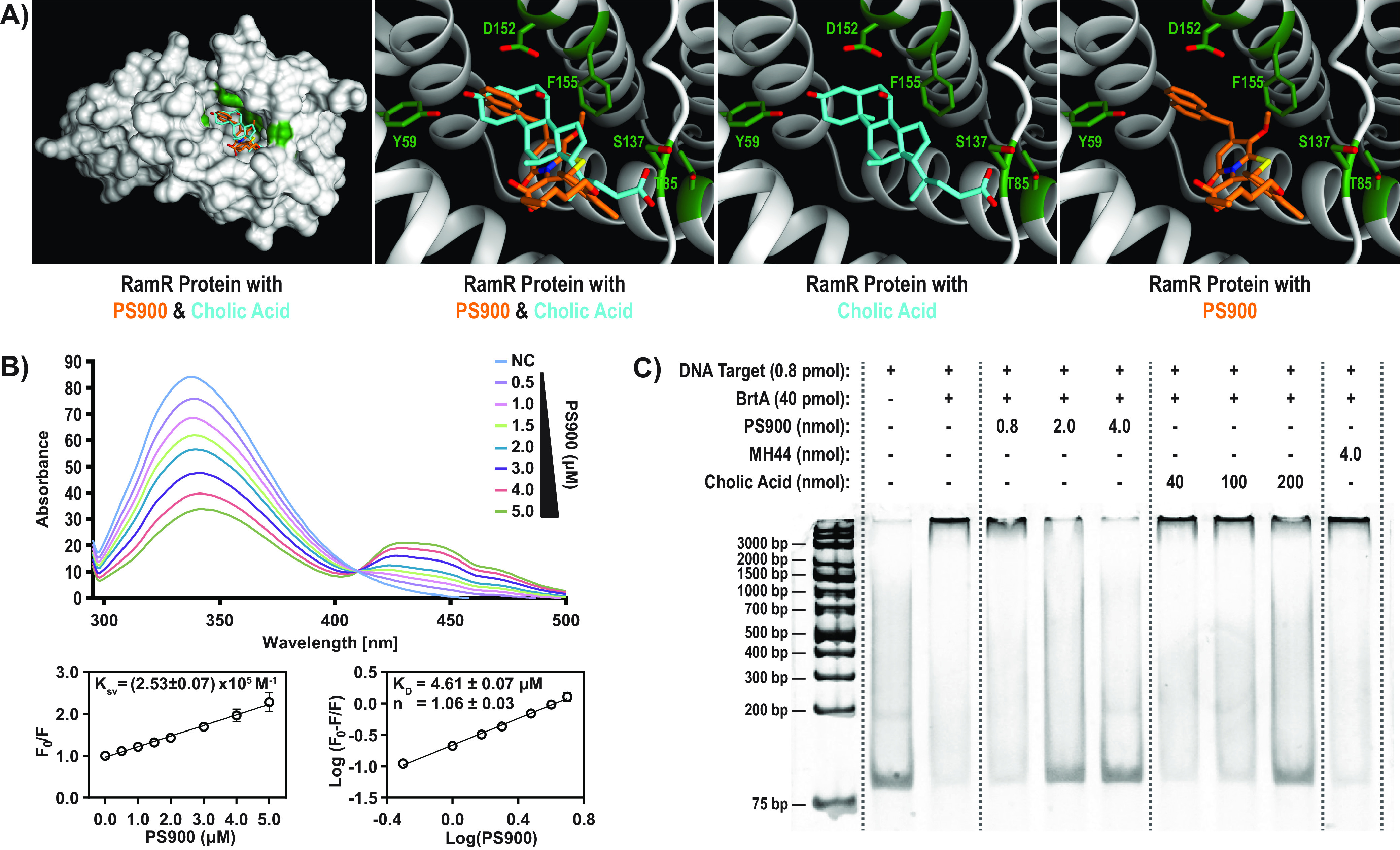
PS900 bind and inactivates the BrtA transcriptiona repressor. (A) The X-ray crystal conformation of cholic acid (cyan) (PDB code 6IE8 [43]) compared to the ldocked conformation of PS900 (orange) within the ligand binding site of the RamR protein from Salmonella enterica serovar Typhimurium. Oxygen, nitrogen, and sulfur atoms are colored red, blue, and yellow, respectively. Potential interaction sites of RamR are highlighted in green. (B) Fluorescence-quenching spectra of BrtA in the presence of PS900. A concentration of 5 μM BrtA in PBS solution was excited at 290 nm, and quenching of fluorescence was recorded in the presence of various concentrations of PS900 (0.5 to 5 μM). Stern-Volmer plots of decreases in fluorescence of BrtA in the presence of various concentrations of PS900 were used to determine the dynamic quenching rate constant, *K*_sv_ (calculated from the slope of the line). Logarithmic plots of relative fluorescence quenching of BrtA against logarithmic concentrations of PS900 were used to determine *K_D_* (calculated from the intersection of the line with the *y* axis) and the number of binding sites, *n* (calculated from the slope of the line). Error bars indicate standard deviations calculated from three individual experiments. (C) EMSA using 85-nucleotide-long DNA fragment comprising the BrtA binding site in front of *mdrT* (0.8 pmol; lanes 2 to 10) (16). Lanes 1 and 2, no protein added. Lanes 3 to 10, binding reaction with 40 pmol BrtA. Lanes 4 to 6, addition of PS900 (0.8, 2.0, or 4.0 nmol). Lanes 7 to 9, addition of cholic acid (40, 100, or 200 nmol). Lane 10, addition of 4 nmol MH44. Lane 1, DNA ladder (Thermo Scientific generuler 1 kb plus, catalog number SM1331).

The simplest explanation for the PS900-mediated increase in *mdrT* expression would be that PS900 is able to interact with and inactivate BrtA in a manner similar to that of CA. We therefore examined whether PS900 could interact directly with BrtA. Superimposing PS900 over CA at the ligand binding site of the BrtA homolog RamR from Salmonella enterica serovar Typhimurium and the QacR homolog *in* Staphylococcus aureus suggested that BrtA could accommodate PS900 (Fig. 4A; Fig. S6). Further support for such a BrtA-PS900 interaction was obtained when examining the ability of PS900 to interact with purified BrtA by using fluorescence quenching (Fig. 4B; Fig. S1). Our results suggested that PS900 and BrtA could interact (*K_D_* [equilibrium dissociation constant] of 4.6 μM), but not MH44 and BrtA. We next asked whether PS900 could displace BrtA from its DNA binding site located upstream from the *mdrT* gene. To test this, electrophoretic mobility shift assays (EMSAs) were conducted in the presence and absence of *mdrT* DNA and the BrtA protein, as well as CA, PS900, or MH44 (Fig. 4C). Whereas the presence of 4 nmol PS900 almost completely disrupted the BrtA-*mdrT* interaction, the same amount of MH44 was unable to affect the complex formation (Fig. 4C). Purified BrtA_F16S_ protein (the same amino acid substitution in BrtA as was identified in the HT014 suppressor strain) bound the operator region more weakly than the BrtA_WT_ protein did (Fig. S7). This was not surprising, considering that the F16S amino acid substitution lies within the HTH DNA binding motif in BrtA (Fig. 2C). The BrtA_F16S_ protein was, however, able to interact as strongly with PS900 as the BrtA_WT_ protein, with a similar *K_D_* (Fig. 4B; Fig. S8). We conclude that PS900 is able to bind and inactivate BrtA, leading to increased *mdrT* expression.

10.1128/mbio.00449-23.6 FIG S6 Predicted docking of PS900 into QacR repressors. Superposition of the docked conformation of PS900 (orange) and cholic acid (cyan) together with the X-ray crystal conformation of rhodamine 6G (purple, PDB code 1JUS) within the ligand binding site of the QacR repressor from Staphylococcus aureus (44). Oxygen, nitrogen, and sulfur atoms are colored red, blue, and yellow, respectively. Download FIG S6, EPS file, 7.8 MB. Copyright © 2023 Tükenmez et al. 2023 Tükenmez et al. https://creativecommons.org/licenses/by/4.0/ This content is distributed under the terms of the Creative Commons Attribution 4.0 International license.

10.1128/mbio.00449-23.7 FIG S7 BrtA protein carrying an F16S point mutation (BrtA^F16S^) binds DNA with a lower efficacy than BrtA^WT^. (A) EMSA using an 85-bp *mdrT* upstream fragment (0.8 pmol; lanes 2 to 10). Lanes 1 and 2, no protein added. Lanes 3 to 10 (left), binding reaction with various concentrations of wild-type BrtA protein (0.8 to 80.0 pmol). Lanes 3 to 10 (right), binding reaction with various concentrations of BrtA^F16S^ protein (0.8 to 80 pmol). Lane 1, DNA ladder (Thermo Scientific generuler 1 kb plus, catalog number SM1331). Efficiency of protein-DNA interaction was determined by quantification of the unmigrated DNA. (B) Plots of decreases in levels of unmigrated DNA in the presence of increasing concentrations of wild-type BrtA (green) or BrtA^F16S^ (blue). All measurements were performed in triplicates, and the results were plotted using GraphPad Prism (version 9.3.1). Black bars represent standard deviations of replicates. Download FIG S7, EPS file, 4.1 MB. Copyright © 2023 Tükenmez et al. 2023 Tükenmez et al. https://creativecommons.org/licenses/by/4.0/ This content is distributed under the terms of the Creative Commons Attribution 4.0 International license.

10.1128/mbio.00449-23.8 FIG S8 Fluorescence-quenching spectra of the BrtA^F16S^ protein in the presence of PS900. A concentration of 5 μM BrtA^F16S^ in PBS solution was excited at 290 nm, and quenching of fluorescence was recorded in the presence of various concentrations of PS900 (0.5 to 5 μM). Stern-Volmer plots of decreases in the fluorescence of BrtA^F16S^ in the presence of various concentrations of PS900 were used to determine the dynamic quenching rate constant, *K*_sv_ (calculated from the slope of the line). Logarithmic plots of relative fluorescence quenching of BrtA^F16S^ against logarithmic concentrations of PS900 were used to determine *K_D_* (calculated from the intersection of the line with the *y* axis) and the number of binding sites, *n* (calculated from the slope of the line). Error bars indicate standard deviations calculated from three individual experiments. Download FIG S8, EPS file, 1.4 MB. Copyright © 2023 Tükenmez et al. 2023 Tükenmez et al. https://creativecommons.org/licenses/by/4.0/ This content is distributed under the terms of the Creative Commons Attribution 4.0 International license.

### Simultaneous addition of CA and PS900 has a bactericidal effect.

**FIG 5:**
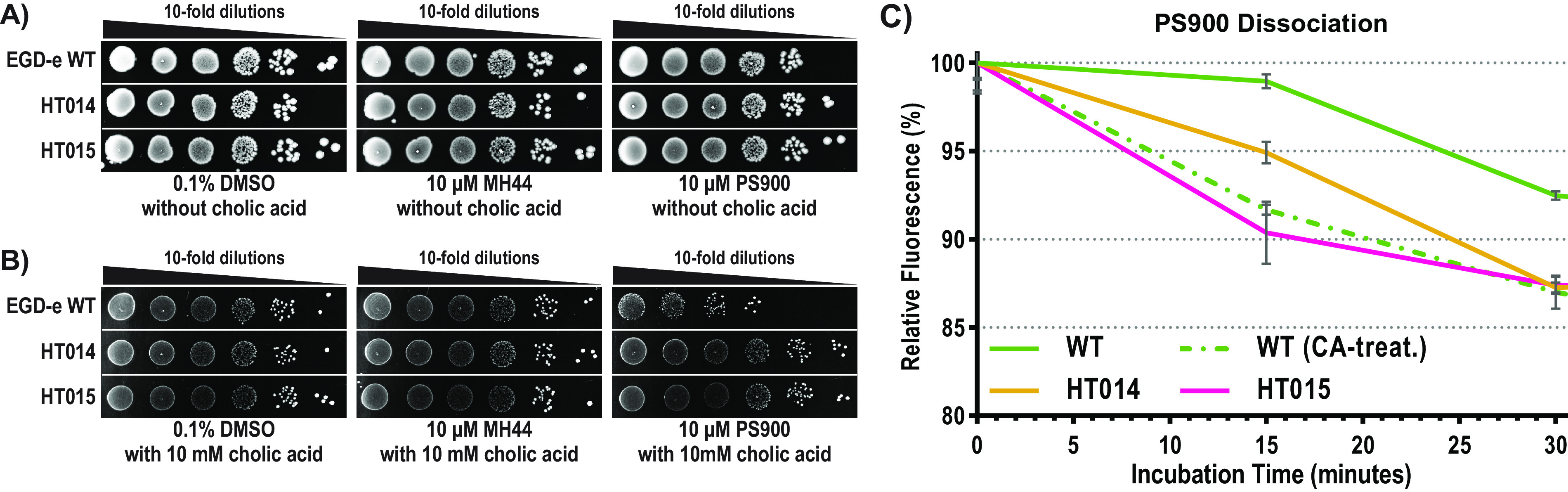
Bactericidal effect of simultaneous addition of cholic acid and PS900. (A) Overnight WT, HT014, and HT015 cultures were serially diluted (10-fold) and then spotted onto BHI agar plates together with 0.1% DMSO (control), 10 μM MH44, or 10 μM PS900 without cholic acid (CA). Growth was visualized after 48 h of incubation at 37°C. (B) Overnight WT, HT014, and HT015 cultures were serially diluted (10-fold) and then spotted onto BHI agar plates together with 0.1% DMSO (control), 10 μM MH44, or 10 μM PS900 with 10 mM cholic acid (CA). Growth was visualized after 48 h of incubation at 37°C. (C) Relative fluorescence of PS900 dissociation from L. monocytogenes WT, HT014, and HT015. Overnight WT, HT014, and HT015 cultures (5 OD unit) were washed and precultivated in BHI medium with/without 10 mM CA for an hour at 37°C. These cultures were treated with 10 μM PS900 for 15 min, washed, and further incubated in PBS for 30 min at 37°C. The fluorescence signals emitted from PS900 (λ_ex_ = 400 nm and λ_em_ = 440 nm) were normalized to the OD_600_ measurements. The dissociation results were plotted as the percentages of PS900 fluorescence (relative to the initial fluorescence, 100% at *t* = 0) over a 30-min time course. All measurements were performed in triplicates, and the results were plotted using GraphPad Prism (version 9.3.1). Error bars represent standard deviations of replicates.

Since PS900 has a bactericidal effect if bacteria are grown in defined medium and it is able to mimic some phenotypes observed after CA addition, we sought to examine whether the addition of PS900 together with CA could also affect bacterial viability in BHI medium (as we see for other Gram-positive pathogens exposed to PS900 [28]). Therefore, WT, HT014, and HT015 bacteria were grown overnight in BHI medium before being spotted onto agar plates containing (or not) 10 μM MH44, 10 μM PS900, or 10 mM CA (Fig. 5A and B). After 48 h of incubation on plates, bacteria grown in the absence of CA or with CA alone showed no difference in viability, whereas bacteria grown in the presence of both CA and PS900 displayed an ~100-fold-reduced viability. In contrast, the growth of strains HT014 and HT015 was unaffected by the simultaneous addition of CA and PS900.

It has previously been shown that the efflux of CA depends on MdrT (16). Our results so far suggest that PS900 efflux also depends on MdrT. To further examine this, we used the autofluorescence properties of PS900 (PS757-IMD) to measure PS900 efflux (28). Our results showed that bacteria not having an induced *mdrT* expression, i.e., not pretreated with CA, had a slow efflux of PS900 (Fig. 5C). In contrast, induced *mdrT* expression, i.e., pretreatment with CA, increased PS900 efflux to a level similar to the efflux observed for the suppressor mutant HT015 (Fig. 5C).

### PS900 sensitizes *Listeria* to different osmolytes.

**FIG 6:**
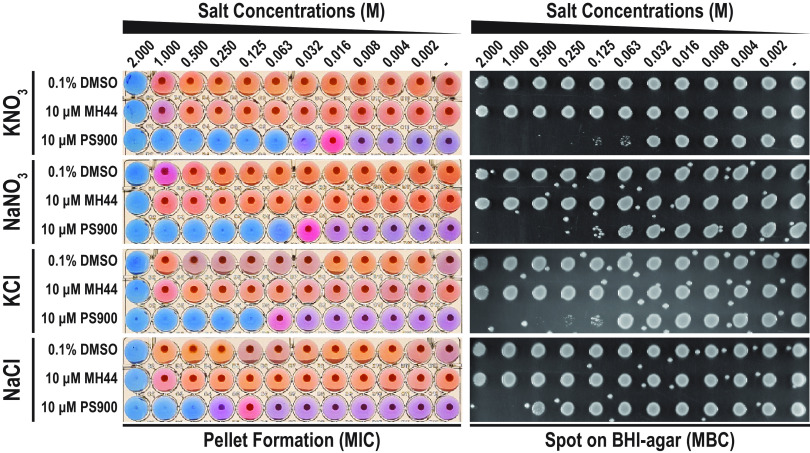
PS900 potentiates sensitivity to osmotic salt stress. Representative images showing the effects of KNO_3_, NaNO_3_, KCl, and NaCl salts on bacterial growth and survival in the presence of DMSO (control), MH44, or PS900. Effects on bacterial growth (MICs) were scored by pellet formation and resazurin (blue)-to-resorufin (pink) conversion (see Materials and Methods). Bacterial survival (MBC) was scored by growth on BHI agar plates. The WT strain was incubated overnight in BHI medium with various concentrations of salts in the presence of 0.1% DMSO (control), 10 μM MH44, or 10 μM PS900 at 37°C. Samples were then spotted onto regular BHI agar plates, followed by 24 h of incubation at 37°C. The images are representative of three individual experiments.

CA is an important part of intestinal bile acid, and L. monocytogenes survival in bile acid has been shown to depend on several osmolyte transporters, such as Gbu, BilE, OpuC, and BilL (20). Considering the additive effect of PS900 on CA-mediated bactericidal activity, we were interested to examine whether PS900 could also sensitize L. monocytogenes to different osmolytes in BHI medium. For this, we examined the viability of bacteria grown in the presence or absence of PS900 and MH44 at different concentrations of KNO_3_, NaNO_3_, KCl, and NaCl (Fig. 6). Strikingly, the addition of 10 μM PS900 decreased bacterial viability already in the presence of 0.063 M KNO_3_. In contrast, growth in the absence of 2-pyridones or in the presence of 10 μM MH44 was unaffected at concentrations of up to 2 M KNO_3_. Similar effects on bacterial growth, although less pronounced, were observed for other osmolytes; when bacteria were exposed simultaneously to PS900 and NaNO_3_, KCl, or NaCl, bacterial growth and viability were reduced (Fig. 6). Interestingly, as a difference from the results of simultaneous exposure to CA and PS900, where the growth of the HT014 and HT015 mutants was unaffected, the same mutant strains were still sensitive to osmolytes (although not as much as the WT strain) (Table S2). This suggests that the osmolyte sensitization in the presence of PS900 could be mediated by a different mechanism than the BrtA/MdrT efflux system.

10.1128/mbio.00449-23.10 TABLE S2 PS900 potentiates sensitivity to osmotic salt stress. Download Table S2, PDF file, 0.04 MB. Copyright © 2023 Tükenmez et al. 2023 Tükenmez et al. https://creativecommons.org/licenses/by/4.0/ This content is distributed under the terms of the Creative Commons Attribution 4.0 International license.

### Metabolite efflux is decreased in bacteria exposed to PS900.

**FIG 7:**
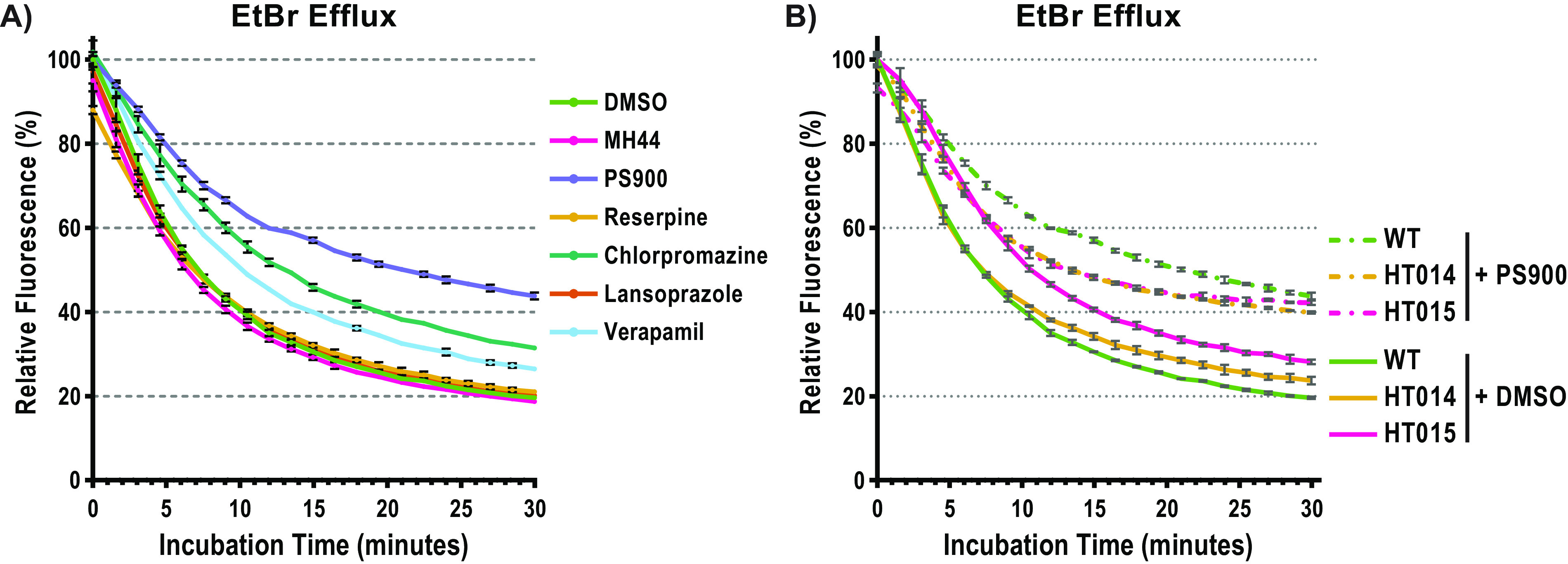
PS900 targets additional efflux pumps. (A) Relative fluorescence of EtBr depletion of L. monocytogenes WT bacteria treated with DMSO (green, vehicle control), MH44 (pink, 10 μM), PS900 (purple, 10 μM), reserpine (yellow, 10 μM), chlorpromazine-HCl (green, 10 μM), lansoprazole (orange, 10 μM), or verapamil (light blue, 10 μM) over a 30-min time course. The results are plotted as the percentages of EtBr relative to the average EtBr levels of the DMSO-treated samples at 0 min. (B) Relative fluorescence of EtBr depletion of L. monocytogenes WT (green), HT014 (yellow), and HT015 (pink) treated with DMSO (solid lines, vehicle control) or PS900 (dotted lines, 10 μM) over a 30-min time course. The results are plotted as the percentages of EtBr relative to the average EtBr levels of the DMSO-treated samples at 0 min. All measurements were performed in triplicates, and the results were plotted using GraphPad Prism (version 9.3.1). Error bars represent standard deviations of replicates.

Why are bacteria killed when they are exposed simultaneously to PS900 and CA or different osmolytes? Based on findings that mutations in the efflux system regulator BrtA allow growth in the presence of PS900 and that very little PS900 is pumped out from the bacteria not having increased *mdrT* expression, we speculated that PS900 would affect bacterial efflux. To test this, we used an efflux assay that measures the general ability of the bacterium to pump out compounds. The assay takes advantage of the fluorescence emitted by ethidium bromide (EtBr) when it interacts with cytoplasmic DNA (33, 34). If not interacting with DNA, EtBr does not emit fluorescence (33, 34). Thus, decreased fluorescence over time is proportional to the rate of overall efflux (28, 33, 34). To follow *de facto* efflux, bacteria were only exposed to compounds at the start of the experiment. When monitoring WT bacteria, we observed that EtBr efflux was efficient in bacteria exposed to MH44, occurring at a level similar to that in the DMSO control (20% fluorescence remaining after 30 min) (Fig. 7A). In contrast, exposing bacteria to PS900 considerably reduced EtBr efflux (~45% remaining after 30 min). In fact, PS900 was more efficient than several commercially available efflux inhibitors, including reserpine (a monoamine transporter inhibitor), lansoprazole (a proton pump inhibitor), and verapamil (a Ca^2+^ channel inhibitor) (Fig. 7A).

To examine whether PS900 also affected the activity of other efflux pumps, we took advantage of the suppressor mutants HT014 and HT015, which show increased *mdrT* expression. If PS900 only targets MdrT, the addition of PS900 to these mutants should reduce EtBr efflux. On the other hand, if PS900 has additional targets, EtBr efflux should remain similar. The addition of PS900 only marginally affected EtBr efflux, indicating that PS900 targets other efflux pumps as well (Fig. 7B).

## DISCUSSION

In this paper, we show that second-generation 2-pyridones substituted with a carbon tail containing an aryl group are able to both repress virulence gene expression (by inactivating the virulence regulator PrfA) (Fig. 1) and potentiate the antibacterial effect of CA, as well as that of osmolytes and nitrite (Fig. 5 and 6). Ring-fused 2-pyridones lacking a carbon tail, here represented by MH44, were identified as antivirulence substances able to attenuate the virulence capacity of L. monocytogenes by binding to an active site in the virulence regulator PrfA (26, 27, 29). This PrfA–2-pyridone interaction displaces PrfA from binding DNA by distorting its HTH motif. Although we observe direct binding between the second-generation 2-pyridones (here represented by PS900) and PrfA and molecular docking experiments suggest that PS900 directly binds to PrfA at the same site as MH44, the exact binding position of PS900 and its effect on PrfA activity remains to be determined.

In contrast to MH44, PS900 displays antibacterial activity (Fig. 2). This is in agreement with a recent study where PS900 was able to kill Gram-positive bacteria of different species, including methicillin-resistant S. aureus (MRSA) and vancomycin-resistant enterococcus (VRE), at low micromolar concentrations (28). However, in contrast to other Gram-positive bacteria, PS900 only shows bactericidal activity for L. monocytogenes in defined medium (DM), and not in BHI medium (Fig. 2). The reason for the difference in phenotypes observed when bacteria were grown in BHI medium or DM is not evident. However, BHI medium contains a plethora of peptides, sugars, and other complex molecules compared to DM. It could thus be speculated that PS900 is sequestered by peptides in BHI, thus inactivating the compound.

We identified two suppressor mutants able to grow in the presence of high PS900 levels (Fig. 2). Whole-genome sequencing showed that both suppressors carried base substitutions in *brtA*, encoding a repressor of the *mdrT* efflux pump, and the levels of *mdrT* were elevated in the suppressor mutants compared with the level in the WT strain. Interestingly, MdrT has been shown to be essential for pumping out CA from the bacterial cytosol (16). CA is a component of bile, and L. monocytogenes uses several mechanisms to survive bile when present in the gut of a host (31). The bacterium is able to colonize the gallbladder, where bile is produced (12). In this study, we show that PS900, like CA, is able to directly interact with and displace BrtA from the *mdrT* promoter region, thereby inducing *mdrT* expression (Fig. 4). Whether PS900 recognizes a similar binding site in BrtA as in PrfA remains to be elucidated.

Our data suggest that PS900 (like CA) uses MdrT for efflux (Fig. 5B). Pretreating the WT bacterium with PS900 or CA allows it to grow in the presence of CA (Fig. 5A), likely due to increased expression of *mdrT* (Fig. 3A; Fig. S4). Since PS900 and CA are bactericidal if added simultaneously to bacteria not having induced *mdrT* expression, we hypothesize that the elevated levels of both chemicals saturate the MdrT pump, thus preventing essential CA efflux. Alternatively, the toxic effect of combining PS900 and CA could be due to the ability of PS900 to effectively decrease overall efflux (Fig. 7A). The presence of both PS900 and CA would lead to a high intracellular concentration of CA or other products, which would be toxic for the cells (16). Furthermore, our results suggest that PS900 can block the activity of other efflux pumps, since the addition of PS900 to the suppressor mutants, which have high levels of *mdrT* expression, does not reduce EtBr efflux (Fig. 7B).

Under hyperosmotic growth, potassium is imported into bacteria to prevent massive water efflux. To prevent disturbances of cellular processes, potassium can be replaced by glycine betaine (35). Interestingly, bile tolerance in L. monocytogenes is dependent on osmolyte transporters like OpuC (a carnitine transporter), BetL (a betaine uptake system), and BilE (a bile exclusion system) (20). In this study, we observed that PS900 sensitized L. monocytogenes to different osmolytes, such as potassium. For instance, PS900 sensitized the bacterium to potassium nitrate (KNO_3_) 50-fold. Since KNO_3_ is important as a preservative in the food industry (E252), our findings identified a role for ring-fused 2-pyridones in combination with other food additives to prevent L. monocytogenes-mediated food spoilage. As for CA, we suggest that the bactericidal effect observed when PS900 was added together with the osmolyte was due to the ability of PS900 to inhibit efflux, thus increasing the intracellular concentration of the osmolyte. Alternatively, PS900 could interact with other targets, such as by affecting the level or activity of c-di-AMP, which is a secondary metabolite controlling the expression of many genes, including osmolyte transporters (36). Also, c-di-AMP binds to OpuC and inactivates it (37).

Novel types of antibacterial agents are desperately needed. We have previously shown that bicyclic thiazolino 2-pyridones are able to block L. monocytogenes virulence (26, 27) and kill Gram-positive pathogens closely related to L. monocytogenes while still not being toxic to human cells (28). Hence, bicyclic 2-pyridone structures constitute an attractive scaffold that can be used in the design of new types of antibacterial agents.

## MATERIALS AND METHODS

### Bacterial strains and growth conditions.

Listeria monocytogenes EGD-e WT strain and PS900-resistant mutants HT014 and HT015 were maintained as frozen stocks (−80°C) in 25% glycerol or as colonies for up to 2 weeks at 4°C on brain heart infusion (BHI) agar (BD Biosciences). Growth experiments were performed at 37°C in BHI or in a chemically defined medium (DM) as previously described (32).

### Synthesis of compounds.

The compounds MH44 and PS900 were synthesized following procedures in the literature (27, 28), and their analytical data were in agreement with those previously reported.

### Fluorescence spectroscopy.

The interaction between proteins (PrfA, BrtA, and BrtA^F16S^) and compounds (MH44 and PS900) was analyzed by the concentration-dependent effect of compounds on the tryptophan fluorescence emission of these proteins, as previously described by Tükenmez et al. (38) with a few minor modifications. The emission spectra of these proteins were acquired using 5 μM protein dissolved in phosphate-buffered saline (PBS) (1×, pH 6.8) with or without compounds in a quartz cuvette. The solutions were excited at 290 nm, and the intrinsic fluorescence emissions were scanned from 295 nm to 500 nm. The results were combined and plotted using Spectragryph software (39). Fluorescence quenching was determined by analyzing the data by the classical Stern-Volmer equation as previously described by Tükenmez et al. (38).

### Molecular docking.

The Protein Preparation Wizard (40, 41) implemented in Maestro (42) was used to prepare the X-ray crystal structures (PDB codes 5F1R [26], 6IE8 [43], and 1JUS [44]) for docking. Ligands, cocrystallizing agents, and water molecules were removed, followed by the addition of hydrogens and optimization of the hydrogen bond network. The receptor grids were prepared based on the positions of the X-ray crystal ligands, resulting in *x*, *y*, and *z* coordinates of 5.74, 1.67, and 16.32 for the structure with PDB code 5F1R, −10.79, −34.65, and 10.90 for the structure with PDB code 6IE8, and −64.53, −46.06, and 3.55 for the structure with PDB code 1JUS. The inner and outer box dimensions were set to 10 Å and 30 Å, respectively.

The ligands were docked using Glide (45–47) implemented in Maestro using standard precision mode. The number of poses included during post-docking minimization was increased to 100 and the number of output poses was increased to 20 per ligand.

### Western blotting.

Overnight cultures of L. monocytogenes were diluted to an optical density at 600 nm (OD_600_) of 0.05 in BHI supplemented with 0.1% DMSO (vol/vol) or 10 μM 2-pyridones (MH44 or PS900) in 96-well plates. The cultures were grown at 37°C with shaking for 5 h to a final OD_600_ of 1.5 to 2.0. The cultures were then centrifuged at 18,000 × *g* for 5 min at room temperature. The supernatants were transferred to new tubes for trichloroacetic acid (TCA) protein precipitation, and the pellets were washed once with 1× PBS. The pellets were suspended with buffer A (200 mM KCl, 50 mM Tris-HCl [pH 8.0], 1 mM EDTA, 10% glycerol, and 1 mM dithiothreitol [DTT]), and ~200 μL glass beads was added. The suspensions were disrupted using a bead-beater for 30 s at maximum speed 3 times and rested on ice for 1 min in between. The suspensions were subsequently centrifuged at 18,000 × *g* for 5 min at 4°C, and supernatants were TCA protein precipitated.

Both supernatant (secreted) and whole-cell lysate fractions were mixed with 1:10 volume of 2% sodium deoxycholate, followed by 10 min of incubation at room temperature. With the addition of a 1:4 volume of ice-cold 50% TCA, the samples were incubated on ice for an hour. Next, the samples were pelleted at 18,000 × *g* for 10 min at 4°C, and the pellets were washed with ice-cold 80% acetone. The dried protein pellets were suspended in 1× Laemmli buffer and boiled at 95°C for 10 min. The samples were then subjected to SDS-PAGE and Western blotting using anti-Bacillus subtilis CodY (ETU005; Kerafast), anti-LLO (ab43018; Abcam), anti-ActA, and anti-PrfA R79IS4b (kindly provided by Pascale Cossart, Institute Pasteur, Paris, France) primary antibodies and a horseradish peroxidase (HRP)-conjugated goat anti-rabbit secondary antibody (as09602; Agrisera). The signal levels were quantified with ImageJ (version 1.53t), and data analysis was done with GraphPad Prism (version 9.4.1).

### Northern blotting.

Overnight cultures of L. monocytogenes were diluted to an OD_600_ of 0.05 in 20 mL BHI medium supplemented with 0.1% DMSO (vol/vol) or 10 μM 2-pyridone (MH44 or PS900) in 100-mL flasks. The cultures were grown at 37°C with shaking for 2.5 to 3 h to an OD_600_ of 0.5 to 0.7. The cultures were split into two subcultures and mixed at a 1:1 ratio with BHI medium supplemented with 2% DMSO (vol/vol) or 20 mM cholic acid, followed by incubation at 37°C with shaking for 30 min. The samples were mixed with 0.2× volume of a 5% phenol–95% ethanol solution and centrifuged at 4,000 × *g* for 15 min at 4°C. The pellets were then stored at −80°C.

Isolation of RNA and Northern blotting were performed as previously described in Oliveira et al. (48), with minor alterations. RNA samples (20 μg from each sample) were run on a 1.2% agarose gel containing 1× HEPES buffer and 7.3% formaldehyde. The RNA samples were then capillary transferred to a Hybond-N membrane (Amersham) and cross-linked by UV light. The membranes were prehybridized in hybri-quick buffer (Roth) for 2 h at 60°C and then hybridized at 60°C overnight together with [^32^P]dATP-α-labeled DNA probes. These DNA probes were prepared using a Prime-a-Gene kit (Promega), and DNA fragments amplified by PCR with corresponding oligonucleotides for the *mdrT* (*lmo2588*) gene (forward, 5′-CCCCAACCATCATTACCCGCTGAACTAAATCCGTATAG-3′, and reverse, 5′-GGTTGGATTGTGGATTCGTATGATTGGCGCG-3′), the *brtA* (*lmo2589*) gene (forward, 5′-CGGCTTCGTTTCCTCCCCGTAAACCTCTAAC-3′, and reverse, 5′-CGCTTGGAGTATACGATGGCGCAGGATGG-3′), and transfer mRNA (tmRNA) (forward, 5′-CCTCGTTATCAACGTCAAAGCC-3′, and reverse, 5′-CGGCACTTAAATATCTACGAGC-3′). Membranes were washed with 0.1% SDS, 2× SSC (1× SSC is 0.15 M NaCl plus 0.015 M sodium citrate) at 65°C for 15 min, followed by washing with 0.1% SDS, 1 × SSC at 65°C for 15 min and 0.1% SDS, 0.5 × SSC. The membranes were exposed in a phosphoscreen cassette, which was then developed using the Typhoon FLA 9500 scanner (GE Healthcare). Signal levels were quantified with ImageQuant 8.2, and data analysis was done with GraphPad Prism (version 9.4.1).

### Genomic DNA sequencing and SNP analysis.

The L. monocytogenes WT (EGD-e), HT014, and HT015 strains were cultivated in BHI medium at 37°C overnight. Genomic DNA from these cultures was prepared using the DNeasy blood & tissue kit (Qiagen) according to the manufacturer’s protocol. Illumina sequencing libraries were prepared by MicrobesNG (Birmingham, United Kingdom) using the Nextera XT library preparation kit (Illumina, USA) according to the manufacturer’s protocol with the following modifications: input DNA was increased 2-fold, and the PCR elongation time was increased to 45 s. Libraries were sequenced with the Illumina NovaSeq 6000 platform using the 250-bp paired-end protocol. The reads were adapter trimmed using Trimmomatic 0.30 with a sliding window quality cutoff score of Q15 (49). SPAdes version 3.7 (50) was used on the samples for *de novo* assembly, and the contigs were annotated using Prokka 1.11 (51). Variants were determined in comparison to the Listeria monocytogenes EDG-e reference genome (ASM19603v1). Details of whole-genome-sequencing (WGS) data and single nucleotide polymorphism (SNP) data can be found in the supplemental material (Table S1).

### EMSA.

Electrophoretic mobility shift assays (EMSAs) were performed with the 85-bp *lmo2588* (*mdrT*) upstream fragment. Complementary oligonucleotides bearing the *mdrT* upstream (−70…+15) sequence were annealed and amplified with oligonucleotides (5′-TGCTGTACTATTCAAAATTAATACCC-3′ and 5′-CTTGTTGGTCTTTTTTTATTATACTTC-3′). The EMSA reactions were performed in 10 mM Tris-HCl (pH 8.0), 150 mM KCl, 0.5 mM EDTA (pH 8.0), 0.1% Triton X-100, 12.5% glycerol, 0.2 mM DTT, 0.1 mg/mL bovine serum albumin (BSA), and 10 ng/μL poly(dI-dC) reaction buffer as previously described in Tükenmez et al. (38). The binding reaction mixtures were set with 0.8 pmol DNA target (85 bp), 40 pmol BrtA protein (WT or F16S variant produced by the Protein Expertise Platform at Umeå University in 1× PBS) with or without the addition of PS900 (0.8, 2.0 or 4.0 nmol), MH44 (4.0 nmol), or cholic acid (40, 100, or 200 nmol). The reactions were incubated for 60 min at 37°C and then were loaded onto 6% polyacrylamide gels and run at 80 V in 0.5% TBE buffer for 70 min. The gels were then stained in 0.5× GelRed (Biotium) buffer for 15 min, followed by washing with Milli-Q water for 15 min thrice. The DNA was visualized with a UV transilluminator, and signals were analyzed by using Image J (version 1.53t).

### EtBr efflux assay.

The EtBr efflux assay was performed as previously described in Nye et al. (28), with minor alterations. L. monocytogenes WT, HT014, and HT015 cultures were grown overnight in BHI medium at 37°C with shaking. EtBr efflux was monitored in the presence of 10 μM MH44, PS900, or another known efflux inhibitor (chlorpromazine-HCl, reserpine, lansoprazole, or verapamil-HCl). All measurements were performed in triplicates, and the fluorescence signals from EtBr were normalized to the average EtBr signal of DMSO (vehicle control)-treated samples at zero minutes.

### PS900 dissociation assay.

The PS900 dissociation assay was performed as previously described in Nye et al. (28), with minor alterations. L. monocytogenes WT, HT014, and HT015 cultures were grown overnight in BHI at 37°C with shaking. Overnight cultures were diluted to an OD_600_ of 1.0 in 5 mL of BHI medium with or without 10 mM CA and incubated for an hour at 37°C. The cultures were then washed once with PBS and normalized to an OD_600_ of 1.0 in 5 mL of PBS. These PBS-washed cells were then incubated with either 0.1% DMSO (vehicle control) or 10 μM PS900 for 15 min at room temperature. The samples were washed once with PBS and then incubated again in PBS for 30 min at 37°C. At 0, 15, and 30 min, 1-mL amounts of these suspensions were harvested by centrifugation, resuspended with 1 mL of PBS, and aliquoted into flat-bottom black 96-well plates with clear bottoms (M0562; Greiner). The fluorescence signals emitted from PS900 (excitation wavelength [λ_ex_] = 400 nm and emission wavelength [λ_em_] = 440 nm), as well as OD_600_ values, were measured with a SpectraMax iD3 microplate reader (Molecular Devices). The fluorescence signals from PS900 were first normalized to the OD_600_ measurements, and then the dissociation results were plotted as the percentages of PS900 fluorescence relative to the initial fluorescence (100%, at *t* = 0) over a 30-min time course. All measurements were performed in triplicates, and the results were plotted using GraphPad Prism (version 9.3.1).

### Impacts of 2-pyridones and osmotic salt stress on bacterial growth and survival.

L. monocytogenes cultures were grown in BHI medium at 37°C with shaking for 5 to 6 h and then diluted to a final OD_600_ of 0.01 in BHI medium supplemented with 0.1% DMSO (vol/vol) or 10 μM 2-pyridones (MH44 or PS900), and various salts (KNO3, NaNO3, KCl or NaCl) were added at various concentrations (0.002 to 2.000 M) in round-bottom 96-well plates. After 18 h of incubation at 37°C, 20 μL (0.1× volume) of 400 μM resazurin (in PBS) was added to each sample for better visualization of the growth, as resazurin (blue dye) is oxidized to resorufin (pink) by living cells. The plates were also centrifuged at 3,500 × *g* for 10 min to score pellet formation as another indication of growth. Treated samples were also spotted onto BHI agar plates, followed by overnight incubation at 37°C to determine survival.

### Data availability.

The raw sequence reads from this study have been deposited in the SRA under accession numbers SRR14598287 (EDGe WT), SRR14598286 (HT014), and SRR14598285 (HT015).
