# Supplementary material for: A Highly Substituted Ring-Fused 2-Pyridone Compound Targeting PrfA and the Efflux Regulator BrtA in Listeria monocytogenes
Source: mBio. 2023 Apr 25;14(3):e00449-23. doi: 10.1128/mbio.00449-23 (PMC10294697; doi:10.1128/mbio.00449-23)
Supplement: TABLE S1 [file mbio.00449-23-s0001.pdf]

Table S1. A. Details of Illumina sequence results

**Trimmed Reads**	**WT**	**HT014**	**HT015**
Median Insert Size	288	350	215
Mean Coverage	118.666	105.964	129.805
Mean Coverage excluding 0s	118.677	105.986	129.814
Number of Reads	930505	808298	1085325
Number of Reads w/ insert size >300	442389	422783	428425

**Assemblies**	**WT**	**HT014**	**HT015**
# contigs	13	14	14
# contigs (>= 0 bp)	35	39	40
# contigs (>= 1000 bp)	11	11	12
# contigs (>= 5000 bp)	8	9	9
# contigs (>= 10000 bp)	8	9	9
# contigs (>= 25000 bp)	8	9	9
# contigs (>= 50000 bp)	7	8	8
Largest contig	886798	886798	886798
Total length	2916594	2916650	2917083
Total length (>= 0 bp)	2921730	2922372	2923339
Total length (>= 1000 bp)	2915393	2914599	2915882
Total length (>= 5000 bp)	2909665	2910022	2910154
Total length (>= 10000 bp)	2909665	2910022	2910154
Total length (>= 25000 bp)	2909665	2910022	2910154
Total length (>= 50000 bp)	2882142	2882499	2882817
N50	583885	583885	583885
N75	496866	425241	424959
L50	2	2	2
L75	4	4	4
GC (%)	37.87	37.87	37.87
**Mismatches**			
# N's	0	0	0
# N's per 100 kbp	0	0	0

Table S1: B. SNP analysis of WT, HT014 & HT015 strains

Chromosome	Position	Locus Tag	Reference Genome*	WT	HT014	HT015	Codon Substitution	Amino Acid Substitution	Mutation Type
AL591824	264578	lmo0247	G	T	T	T	ggG/ggT	G49	SILENT
AL591824	435968	-	C	A	A	A	-	-	-
AL591824	1442124	-	C	A	A	A	-	-	-
AL591824	2943565	rpmH	G	T	T	T	-	-	INTRAGENIC
AL591824	2672465	lmo2589	A	A	G	A	tTt/tCt	F16S	MISSENSE
AL591824	2672151	lmo2589	T	T	T	TC	ctg/ctgG	L120L?	+1 FRAMESHIFT
AL591824	1581346	mreC	A	A	A	G	Tac/Cac	Y116H	MISSENSE
AL591824	929961	rsbV	G	G	G	T	Gat/Tat	D24Y	MISSENSE

*** Reference Genome:** GCA_000196035.1 (ASM19603v1)
