# Supplementary material for: A Highly Substituted Ring-Fused 2-Pyridone Compound Targeting PrfA and the Efflux Regulator BrtA in Listeria monocytogenes
Source: mBio. 2023 Apr 25;14(3):e00449-23. doi: 10.1128/mbio.00449-23 (PMC10294697; doi:10.1128/mbio.00449-23)
Supplement: TABLE S2 [file mbio.00449-23-s0002.pdf]

**Table S2.** PS900 potentiates sensitivity to osmotic salt stress.

| | | $NaNO_3$ | | $KNO_3$ | | NaCl | | KCl | |
|---|---|---|---|---|---|---|---|---|---|
| MIC Values | | MIC* | MBC** | MIC* | MBC** | MIC* | MBC** | MIC* | MBC** |
| WT | 0.1% DMSO | 2.00 | >2.00 | 2.00 | >2.00 | 2.00 | >2.00 | 2.00 | >2.00 |
| | 10 μM MH44 | 2.00 | >2.00 | 2.00 | >2.00 | 2.00 | >2.00 | 2.00 | >2.00 |
| | 10 μM PS900 | 0.25-0.50 | 1.00 | 0.06-0.25 | 0.25-0.50 | 0.25-0.50 | 0.50-1.00 | 0.12-0.25 | 0.12-0.25 |
| HT014 | 0.1% DMSO | 2.00 | >2.00 | 2.00 | >2.00 | 2.00 | >2.00 | 2.00 | >2.00 |
| | 10 μM PS900 | 1.00-2.00 | 2.00 | 0.25-0.50 | 0.25-1.00 | 0.20-1.00 | 2.00 | 0.25-0.50 | 0.50-1.00 |
| HT015 | 0.1% DMSO | 2.00 | >2.00 | 2.00 | >2.00 | 2.00 | >2.00 | 2.00 | >2.00 |
| | 10 μM PS900 | 2.00 | 2.00 | 0.50-1.00 | 0.50-1.00 | 2.00 | 2.00 | 0.50-1.00 | 0.50-1.00 |

** Effect of bacterial growth (MIC) scored by pellet formation and resazurin (blue) to resorufin (pink) conversion (see Figure 6, Materials & Methods).

** Bacterial survival (MBC) scored by growth on BHI-agar plates (see Figure 6, Materials & Methods).

## References:


1. Jumper J, Evans R, Pritzel A, Green T, Figurnov M, Ronneberger O, Tunyasuvunakool K, Bates R, Zidek A, Potapenko A, Bridgland A, Meyer C, Kohl SAA, Ballard AJ, Cowie A, Romera-Paredes B, Nikolov S, Jain R, Adler J, Back T, Petersen S, Reiman D, Clancy E, Zielinski M, Steinegger M, Pacholska M, Berghammer T, Bodenstein S, Silver D, Vinyals O, Senior AW, Kavukcuoglu K, Kohli P, Hassabis D. 2021. Highly accurate protein structure prediction with AlphaFold. Nature 596:583-589.

2. Varadi M, Anyango S, Deshpande M, Nair S, Natassia C, Yordanova G, Yuan D, Stroe O, Wood G, Laydon A, Zidek A, Green T, Tunyasuvunakool K, Petersen S, Jumper J, Clancy E, Green R, Vora A, Lutfi M, Figurnov M, Cowie A, Hobbs N, Kohli P, Kleywegt G, Birney E, Hassabis D, Velankar S. 2022. AlphaFold Protein Structure Database: massively expanding the structural coverage of protein-sequence space with high-accuracy models. Nucleic Acids Res 50:D439-D444.

3. Schumacher MA, Miller MC, Grkovic S, Brown MH, Skurray RA, Brennan RG. 2001. Structural mechanisms of QacR induction and multidrug recognition. Science 294:2158-63.
